# *Wolbachia* Blocks Currently Circulating Zika Virus Isolates in Brazilian *Aedes aegypti* Mosquitoes

**DOI:** 10.1016/j.chom.2016.04.021

**Published:** 2016-06-08

**Authors:** Heverton Leandro Carneiro Dutra, Marcele Neves Rocha, Fernando Braga Stehling Dias, Simone Brutman Mansur, Eric Pearce Caragata, Luciano Andrade Moreira

**Affiliations:** 1 Mosquitos Vetores: Endossimbiontes e Interação Patógeno-Vetor, Centro de Pesquisas René Rachou—Fiocruz, Belo Horizonte, MG, 30190-002, Brazil; 2 Plataforma de Vetores de Doenças—Fiocruz, CE, 60190-800, Brazil

## Abstract

The recent association of Zika virus with cases of microcephaly has sparked a global health crisis and highlighted the need for mechanisms to combat the Zika vector, *Aedes aegypti* mosquitoes. *Wolbachia pipientis*, a bacterial endosymbiont of insect, has recently garnered attention as a mechanism for arbovirus control. Here we report that *Aedes aegypti* harboring *Wolbachia* are highly resistant to infection with two currently circulating Zika virus isolates from the recent Brazilian epidemic. *Wolbachia*-harboring mosquitoes displayed lower viral prevalence and intensity and decreased disseminated infection and, critically, did not carry infectious virus in the saliva, suggesting that viral transmission was blocked. Our data indicate that the use of *Wolbachia*-harboring mosquitoes could represent an effective mechanism to reduce Zika virus transmission and should be included as part of Zika control strategies.

## Highlights

• Mosquitoes harboring *Wolbachia* were resistant to current circulating Zika virus isolates
• Zika virus prevalence, intensity, and disseminated infection were reduced
• Saliva from *Wolbachia*-harboring mosquitoes did not contain infectious Zika virus

## Graphical Abstract

**Figure:**
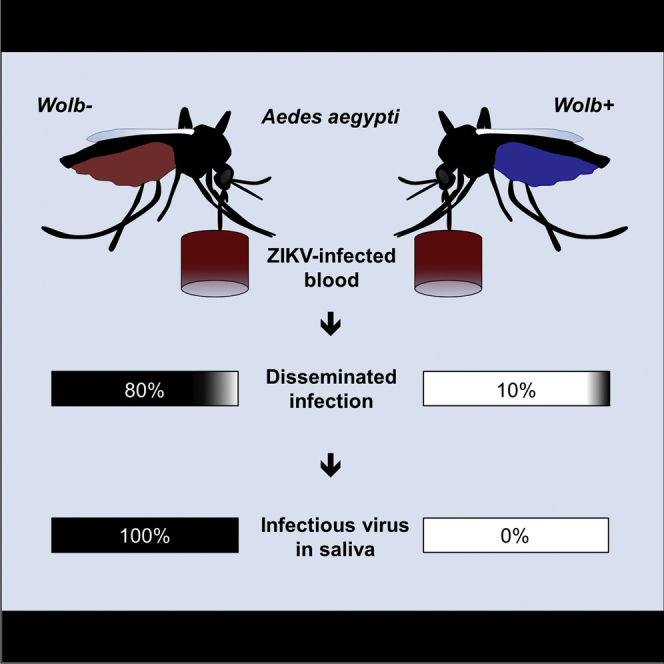

## Teaser

Strategies to combat Zika virus (ZIKV) and its mosquito vector are urgently needed. Dutra et al. report that *Wolbachia*-carrying mosquitoes are highly resistant to ZIKV and display reduced virus prevalence and intensity. Saliva from *Wolbachia*-carrying mosquitoes did not contain infectious virus, suggesting the possibility to block ZIKV transmission.

## Main Text

The mosquito *Aedes aegypti*, typically linked with dengue (*Flaviviridae*) (Kyle and Harris, 2008) and chikungunya (*Togaviridae*) (Morrison, 2014) transmission, is also associated with the alarming spread of Zika virus (ZIKV) (*Flaviviridae*), a previously obscure arbovirus that has recently gone global (Enserink, 2015). Since 2007, ZIKV infection has been reported in 39 countries worldwide (Martínez de Salazar et al., 2016), including Brazil, where infection was first linked to cases of microcephaly during a large outbreak in 2015 (Mlakar et al., 2016, Oliveira Melo et al., 2016). Combined with the implication of the virus in cases of the autoimmune disorder Guillain-Barré syndrome (Araujo et al., 2016), ZIKV has ballooned into a public health crisis.

In the absence of a vaccine, current effective control options are limited to reducing the abundance of mosquito vector populations (Heintze et al., 2007). However, there is a clear need for novel efficacious approaches, given that existing strategies such as insecticides (Maciel-de-Freitas et al., 2014) and larval biological control (Vu et al., 2005) have proven unsustainable and ineffective at halting disease spread (Kyle and Harris, 2008).

After decades of being proposed as a potential means of vector control, the endosymbiotic bacterium *Wolbachia*, present in an estimated 40% of all known terrestrial insect species (Zug and Hammerstein, 2012), is currently being utilized around the world as part of an innovative approach to control the transmission of dengue (http://www.eliminatedengue.com) and other pathogens (Bourtzis et al., 2014). This is possible because the reproductive parasitism associated with *Wolbachia* infection, typified by cytoplasmic incompatibility (Werren et al., 2008), gives the bacterium the ability to quickly and stably invade host populations (Hoffmann et al., 2011). Critically, the bacterium also blocks the transmission of many important human pathogens in mosquitoes, including *Plasmodium* and chikungunya (Bian et al., 2013, Caragata et al., 2016, Moreira et al., 2009), giving it great utility as a control agent.

As many different strains of the bacterium cause this inhibition, we hypothesized that the *w*Mel *Wolbachia* strain (*w*Mel_Br), currently being utilized as part of dengue control efforts in Brazil, might be able to restrict ZIKV infection and transmission in *Ae. aegypti*. To that end, we performed experimental infections with two currently circulating ZIKV isolates and used a qRT-PCR-based assay to a quantify ZIKV levels in mosquito tissues and saliva, in order to assess whether *Wolbachia* could potentially be used to combat the emerging Zika pandemic.

Through experimental infection and transmission assays using two currently circulating Brazilian ZIKV isolates (BRPE243/2015 [BRPE] and SPH/2015 [SPH]) (Faria et al., 2016), we compared ZIKV infection in *w*Mel-infected mosquitoes (*w*Mel_Br) with *Wolbachia*-uninfected mosquitoes collected in Urca, Rio de Janeiro, Brazil in early 2016 (Br). Due to the regular introduction of F_1_ Br males (the eggs of field-collected Br mosquitoes) in *w*Mel_Br colony cages over 2 years, both lines had a similar genetic background (see Supplemental Experimental Procedures).

**Figure 1:**
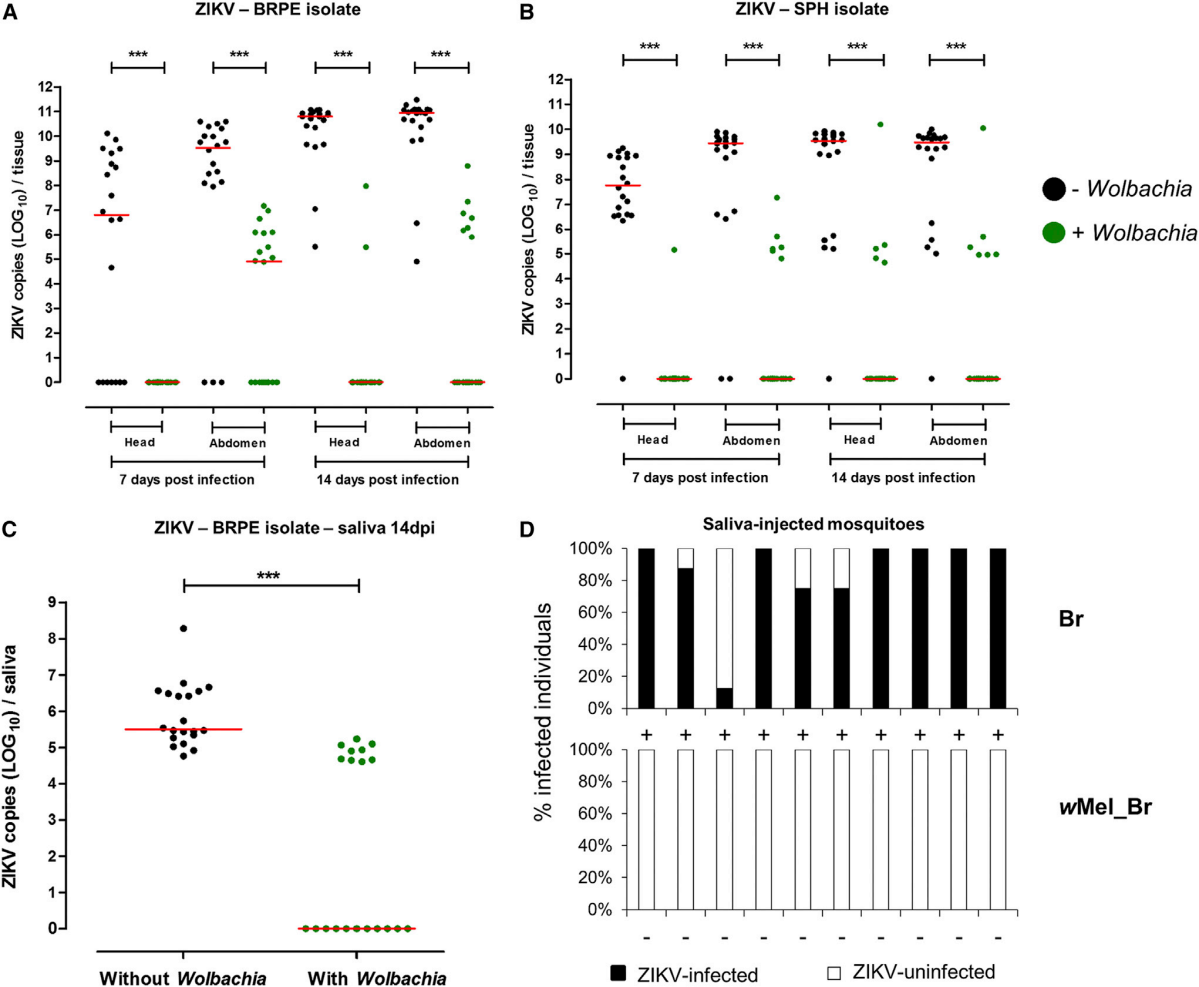
*Wolbachia* Infection Restricts ZIKV Infection in *Ae*. *aegypti* Mosquitoes (A–C) *Wolbachia*-infected (green circles) and -uninfected (black circles) mosquitoes were orally challenged with either (A) the BRPE or (B) the SPH ZIKV isolates. *Wolbachia* infection reduced both prevalence and intensity of ZIKV infection in mosquito heads/thoraces and abdomens at 7 and 14 dpi. Saliva was then collected for mosquitoes infected with the BRPE ZIKV isolate at 14 dpi infection (C), and we observed that saliva from *Wolbachia*-infected mosquitoes had a significantly lower rate of saliva infection and median viral load. (D) When these saliva samples were injected into ZIKV-uninfected Br mosquitoes, all of the Br saliva samples contained infectious virus, while no *w*Mel_Br saliva produced a subsequent infection (columns: black, percentage infected; white, percentage uninfected; +, saliva contained infectious virus, −, saliva did not contain infectious virus). Absolute ZIKV copy numbers were quantified via qRT-PCR. In (A)–(C), each circle represents tissue or saliva from a single adult female (n = 20 per group). Red lines indicate the median ZIKV copies. ^∗∗∗^, p < 0.0001; analysis by Mann-Whitney U test. In (D), each column represents mosquitoes injected with a single saliva sample.

The ZIKVs were isolated in the field in late 2015 and maintained in cell culture, and viral titers were quantified via plaque-forming assay prior to experimental infection (Table 1). In two separate experiments, fresh ZIKV-infected supernatant was harvested from culture, mixed with human blood, and used to orally infect *w*Mel_Br and Br mosquitoes. ZIKV levels were quantified in mosquito heads/thoraces and in abdomens at 7 and 14 days post-infection (dpi) using a TaqMan-based qRT-PCR assay (Figure 1).

The prevalence of ZIKV infection was significantly reduced among *Wolbachia*-infected mosquitoes (Table 1, analysis via Fisher’s exact test, p < 0.0001 unless stated). For the BRPE isolate (Figure 1A), *Wolbachia* decreased ZIKV prevalence by 35% in abdomens, although there was no significant difference for this tissue (p > 0.05), by 100% in head/thoraces at 7 dpi, and by 65% and 90% at 14 dpi, respectively. For the SPH isolate (Figure 1B), *Wolbachia* reduced prevalence by 95% and 67% in head/thoraces and abdomens (p = 0.0002), respectively, at 7 dpi, and by 74% and 68% in head/thoraces and abdomens, respectively, at 14 dpi.

Likewise, the intensity of ZIKV infection was greatly reduced in *w*Mel_Br mosquitoes for both tissues and time points (Mann-Whitney U tests, p < 0.0001). Additionally, we observed that median ZIKV titers in the head/thoraces of Br mosquitoes increased over time for both isolates (Mann-Whitney U test; BRPE, p < 0.0001; SPH, p = 0.0094), while there was no such effect in *w*Mel_Br mosquitoes.

Saliva was collected from Br and *w*Mel_Br mosquitoes at 14 dpi, after the 5- to 10-day ZIKV extrinsic incubation period was likely completed (Li et al., 2012), in order to determine if *Wolbachia* infection also inhibited ZIKV transmission (Figure 1C). We used mosquitoes infected with the BRPE isolate as it had a higher titer in culture (Table 1). ZIKV levels were quantified directly for individual saliva samples using the same qRT-PCR assay. We observed that *Wolbachia* infection reduced ZIKV prevalence in individual saliva samples by 55% (Fisher’s exact test, p < 0.0001) and median ZIKV copies by approximately 5 logs (Mann-Whitney U test, p < 0.0001).

To determine if the virus in these samples was infectious, a further ten *w*Mel_Br and ten Br saliva samples, from the samples described above, were intrathoracically injected into 8–14 naive Br mosquitoes each (Figure 1D), using a previously described method (Ferguson et al., 2015). The overall mortality rate among injected mosquitoes was 11.93%. The presence or absence of ZIKV infection was determined at 5 dpi in eight mosquitoes injected with each saliva, amounting to a mean proportion sampled of 0.68. Of the 80 mosquitoes injected with Br saliva, 68 (85%) became infected with ZIKV, with all Br saliva samples producing at least one infected mosquito. In contrast, none of the 80 mosquitoes injected with *w*Mel_Br saliva became infected (Fisher’s exact test, p < 0.0001; odds ratio 882.3, 95% CI, 51.3–15187), indicating that while some of the *w*Mel_Br saliva samples did contain detectable ZIKV, we saw no evidence that the saliva contained infectious virus.

There is a clear correlation between the inhibition of pathogens by *Wolbachia* and bacterial density in insect tissues (Joubert et al., 2016, Martinez et al., 2014). In order to determine if there was a link between *Wolbachia* density and ZIKV prevalence and intensity, we measured total *Wolbachia* RNA levels in the *w*Mel_Br mosquitoes used in the ZIKV infection assays, using qRT-PCR as described above. We saw that ZIKV infection explained less than 5% of the variance in *Wolbachia* density that was observed between ZIKV-infected and -uninfected *w*Mel_Br mosquitoes at either 7 dpi or 14 dpi and was not a significant predictor (PERMANOVA; p > 0.05). Furthermore, we observed no relationship between *Wolbachia* density and ZIKV load among *w*Mel_Br mosquitoes that became infected with the virus (Spearman correlation; heads/thoraces, *r* = 0.5952, p = 0.1323; abdomens, *r* = −0.01891, p = 0.9210). This suggests that there may not be a direct link between *Wolbachia* density in individual mosquitoes and ZIKV infection, indicating that the inhibition of ZIKV may arise through other means, indirectly due to the presence of the bacterium (Caragata et al., 2013, Moreira et al., 2009, Pan et al., 2012, Rancès et al., 2012).

Our results indicate that the ability of *Wolbachia* infection to greatly reduce the capacity of mosquitoes to harbor and transmit a range of medically important pathogens, including the dengue and chikungunya viruses (Caragata et al., 2016, Moreira et al., 2009, Walker et al., 2011) also extends to ZIKV. While *w*Mel did not completely inhibit ZIKV infection, we observed a similar decrease in prevalence and intensity of infection to that of *w*Mel-infected *Ae. aegypti* challenged with viremic blood from dengue patients, which was considered sufficient to drastically decrease viral transmission (Ferguson et al., 2015). Additionally, the fact that we did not observe an increase in disseminated ZIKV infection over time, and that ZIKV prevalence and infectivity in *w*Mel_Br mosquito saliva was significantly decreased, may indicate that, as for dengue, *w*Mel extends the ZIKV extrinsic incubation period (Ye et al., 2015). This in turn would likely further decrease overall ZIKV transmission rates, given the small decrease in lifespan associated with *w*Mel infection (Walker et al., 2011).

We observed that the *w*Mel *Wolbachia* infection in *Ae. aegypti* greatly inhibited ZIKV infection in mosquito abdomens, and it reduced disseminated infection in heads and thoraces and ZIKV prevalence in mosquito saliva. Most critically, our results suggest that saliva from *w*Mel-infected mosquitoes did not contain infectious virus. That this inhibition occurred for two ZIKV isolates that circulated in Brazil during the 2015 epidemic, and for mosquitoes with a wild-type genetic background, suggests that *w*Mel could greatly reduce ZIKV transmission in field populations of *Ae. aegypti*, which in turn would likely reduce the frequency of Zika-associated pathology in humans.

*Wolbachia* can invade and persist in wild mosquito populations (Hoffmann et al., 2014) and represents a relatively low-cost, self-sustaining form of mosquito control that is already being trialed in countries where ZIKV outbreaks have been reported and has recently been recommended by the World Health Organization as a suitable tool to control ZIKV transmission (http://migre.me/tDWVe). It is important to point out that extensive public engagement will be required before releases of *Wolbachia*-infected mosquitoes can be scaled up for use in other areas. However, the results presented here indicate that *w*Mel-infected *Ae. aegypti* represent a realistic and effective option to combat the ZIKV burden in Brazil and potentially in other countries and should be considered as an integral part of future control efforts.

The work reported in this paper was performed under the oversight of the Committee for Ethics in Research (CEP)/FIOCRUZ (License CEP 732.621).

## Author Contributions

Conceptualization, H.L.C.D., M.N.R., and L.A.M.; Methodology, H.L.C.D. F.B.S.D., E.P.C., and L.A.M.; Formal analysis, H.L.C.D. and E.P.C.; Investigation, H.L.C.D.; M.N.R., F.B.S.D., S.B.M., and E.P.C.; Writing—Original Draft, H.L.C.D.; Writing—Review & Editing, H.L.C.D., E.P.C., and L.A.M.; Funding Acquisition, L.A.M; Resources, L.A.M.; Supervision, L.A.M.

## Figures and Tables

**Table 1:** Effects of *Wolbachia* on ZIKV Prevalence

Isolate	ZIKV Titer (PFU/mL)	Days Post-infection	*w*Mel_Br	Br	*w*Mel_Br	Br	*w*Mel_Br	Br
Head/Thorax Infection Rate		Abdomen Infection Rate		Saliva Infection Rate	
BRPE	5.0 × 10^6^	7	0	65	55	85	–	–
14	10	100	35	100	45	100
SPH	8.7 × 10^3^	7	5	95	30	90	–	–
14	25	95	30	95	–	–

*Ae. aegypti* were orally infected with fresh, low-passage ZIKV. Initial viral titer was determined by plaque-forming assay. Saliva infection was only examined for mosquitoes at 14 days post-infection with the BRPE isolate. Infection rates are given as percentages. n = 20 per group unless specified. ZIKV, Zika virus; PFU, plaque-forming units; BRPE, ZIKV/*H. sapiens*/Brazil/BRPE243/2015; SPH, ZIKV/*H. sapiens*/Brazil/SPH/2015; *w*Mel_Br, *Wolbachia*-infected; Br, *Wolbachia-*uninfected.

## Supplemental Information

Document S1. Supplemental Experimental Procedures and Tables S1–S4

Document S2. Article plus Supplemental Information
