# Supplementary material for: Wolbachia Blocks Currently Circulating Zika Virus Isolates in Brazilian Aedes aegypti Mosquitoes
Source: Cell Host Microbe. 2016 Jun 8;19(6):771–4. doi: 10.1016/j.chom.2016.04.021 (PMC4906366; doi:10.1016/j.chom.2016.04.021)
Supplement: Document S1. Supplemental Experimental Procedures and Tables S1–S4 [file mmc1.pdf]

Cell Host & Microbe, Volume *19*

Supplemental Information

Wolbachia Blocks Currently Circulating Zika Virus Isolates in Brazilian *Aedes aegypti* Mosquitoes

Heverton Leandro Carneiro Dutra, Marcele Neves Rocha, Fernando Braga Stehling Dias, Simone Brutman Mansur, Eric Pearce Caragata, and Luciano Andrade Moreira

Table S1, related to Figure 1. Statistical output for comparison of ZIKV levels in *Wolbachia*-infected and uninfected mosquito tissues

***w*Mel_Br vs Br**	**Infection prevalence: Fisher's exact test**	**Infection intensity: Mann Whitney U test**
**BRPE**		
7dpi heads/thoraces	$P < 0.0001$	$U = 70.00$, $P < 0.0001$
7dpi abdomens	$P = 0.0824$	$U = 46.50$, $P < 0.0001$
14dpi heads/thoraces	$P < 0.0001$	$U = 2.00$, $P < 0.0001$
14dpi abdomens	$P < 0.0001$	$U = 11.00$, $P < 0.0001$
**SPH**		
7dpi heads/thoraces	$P < 0.0001$	$U = 10.50$, $P < 0.0001$
7dpi abdomens	$P = 0.0002$	$U = 29.00$, $P < 0.0001$
14dpi heads/thoraces	$P < 0.0001$	$U = 34.50$, $P < 0.0001$
14dpi abdomens	$P < 0.0001$	$U = 37.00$, $P < 0.0001$

Abbreviations: 7/14dpi - 7/14 days post infection. BRPE - ZIKV/*H. sapiens*/Brazil/BRPE243/2015, SPH - ZIKV/*H. sapiens*/Brazil/SPH/2015, *w*Mel_Br - *Wolbachia*-infected *Ae. aegypti*, Br - *Wolbachia*-uninfected *Ae. aegypti*.

Table S2, related to Figure 1. Statistical output for comparison of ZIKV levels in mosquito tissues over time.

**7dpi vs 14dpi**	**Mann Whitney U test**
**BRPE**	
*w*Mel_Br heads/thoraces	$U = 180.0$, $P = 0.1626$
wMel_Br abdomens	$U = 182.5$, $P = 0.6146$
Br heads/thoraces	$U = 27.00$, $P < 0.0001$
Br abdomens	$U = 52.00$, $P < 0.0001$
**SPH**	
*w*Mel_Br heads/thoraces	$U = 159.5$, $P = 0.0816$
wMel_Br abdomens	$U = 199.0$, $P = 0.9867$
Br heads/thoraces	$U = 103.5$, $P = 0.0094$
Br abdomens	$U = 189.0$, $P = 0.7764$

Abbreviations: 7/14dpi - 7/14 days post infection. BRPE - ZIKV/*H. sapiens*/Brazil/BRPE243/2015, SPH - ZIKV/*H. sapiens*/Brazil/SPH/2015, *w*Mel_Br - *Wolbachia*-infected *Ae. aegypti*, Br - *Wolbachia*-uninfected *Ae. aegypti*.

Table S3, related to Figure 1. Statistical output for comparison of ZIKV levels in the saliva of *Wolbachia*-infected and -uninfected mosquitoes

***w*Mel_Br vs Br saliva**	**Infection prevalence: Fisher's exact test**	**Infection intensity: Mann Whitney U test**
Individual saliva samples	$P = 0.0001$	$U = 13.00$, $P < 0.0001$
Saliva-injected mosquitoes	$P < 0.0001$	NA

Abbreviations: *w*Mel_Br - *Wolbachia*-infected *Ae. aegypti*, Br - *Wolbachia*-uninfected *Ae. aegypti*.

Table S4, related to Figure 1. Statistical output for comparison of *Wolbachia* density amongst ZIKV-infected and -uninfected *w*Mel_Br mosquitoes

PREMANOVA

**7dpi**	**df**	**SS**	**MS**	F	R^2	**Pr(>F)**
ZIKV infection	1	2.61E+14	2.61E+14	1.8286	0.04434	0.186
ZIKV isolate	1	6.31E+14	6.31E+14	4.4139	0.10702	0.029
Residuals	35	5.00E+15	1.43E+14		0.84864	
Total	37	5.90E+15			1.00000	

**14dpi**	**df**	**SS**	**MS**	F	R^2	**Pr(>F)**
ZIKV infection	1	5.63E+13	5.63E+13	0.1188	0.00289	0.813
ZIKV isolate	1	1.85E+15	1.85E+15	3.9087	0.09527	0.053
Residuals	37	1.76E+16	4.74E+14		0.90184	
Total	39	1.95E+16			1.00000	

Abbreviations: ZIKV - Zika virus, 7/14dpi - 7/14 days post infection.

Supplemental Experimental Procedures

Mosquito rearing

All experiments involved two *Ae. aegypti* lines. The first (*w*Mel_Br) was generated by introducing the *w*Mel *Wolbachia* strain into a Brazilian genetic background through backcrossing (Dutra et al., 2015). Experiments were performed 35 generations after the initial backcrossing. The second, (Br) was an F_1 wild-type line derived from material collected from ovitraps in the suburb of Urca, RJ, Brazil at the beginning of 2016. This line never had any contact with *Wolbachia*-infected mosquitoes. For 25 generations prior to experimentation, 200 F_1-F_2 Br males for every 600 *w*Mel_Br females were introduced into *w*Mel_Br colony cages each generation to prevent inbreeding effects, and maintain a similar genetic background between the two lines. All mosquitoes were maintained in a climate-controlled insectary under previously described conditions (Dutra et al., 2015).

ZIKV isolation and culture

The Zika viruses used in this work were isolated in 2015 from human serum collected from two symptomatic patients, the first one from Recife, PE, in northeastern Brazil (ZIKV/*H. sapiens*/Brazil/BRPE243/2015), and the second from Sumaré, SP, in the Southeast of the country (ZIKV/*H. sapiens*/Brazil/SPH/2015) (Faria et al., 2016). Virus stocks were passaged in *Aedes albopictus* cell line (C6/36) grown in Leibowitz L-15 medium supplemented with 10% fetal bovine serum (FBS), 1% penicillin/streptomycin (Gibco), and maintained at 28ºC, as previously described (Hamel et al., 2015). Fresh supernatant from infected C6/36 cells was harvested 7 days after infection with a corresponding viral titer of $5x10^6$ PFU/mL for the BRPE isolate, and $8.7x10^3$ PFU/mL for the SPH isolate, and used to orally infect mosquitoes without ever being frozen.

Infection of mosquitoes with ZIKV

ZIKV was collected from C6/36 cell culture supernatant and then re-suspended 2:1 in fresh whole human blood. Four days-old adult female mosquitoes were starved for 24 hrs prior to feeding, and allowed to feed on the blood-virus mixtures for 1 hr using glass feeders covered with pig intestine as a membrane, and maintained at 37°C using a water bath. After feeding, mosquitoes that were not fully engorged were removed. Mosquitoes were collected at both 7dpi and 14dpi, and stored at -80ºC until processing.

Saliva collection

Individual mosquito saliva was collected at 14 days post-infection from mosquitoes infected with the BRPE ZIKV isolate according to a previously published protocol, with some modifications (Anderson et al., 2010). Briefly, mosquitoes were starved overnight prior to harvesting. On collection day, mosquitoes were knocked down with CO_2, and kept at 4ºC while legs and wings were removed. Each mosquito's proboscis was inserted into a sterile, filtered 10µL pipette tip containing 5µL of a 1:1 solution of sterile fetal bovine serum: 30% sucrose, and allowed to salivate for 30 minutes. Mosquitoes were then visually verified to be alive by checking for movement. The contents of the tips were then collected in sterile 0.5mL tubes and stored at -80ºC prior to processing. One third of the saliva samples were used for injections while the remainder were used for direct quantification.

Confirmation of saliva ZIKV infectivity

Female Br mosquitoes were injected intrathoracically with saliva collected from ZIKV-infected *w*Mel_Br or Br females, in order to determine if the saliva contained infectious virus. Mosquitoes were injected using a Nanoject II hand held injector (Drummond), as previously described (Moreira et al., 2009). Each saliva sample was used to inoculate between 8-14 mosquitoes, with each receiving an average of 276nL. To avoid contamination, a fresh needle was used for each saliva. Mosquitoes were collected 5 dpi, and the presence or absence of virus was determined by RT-qPCR screening of 8 individual mosquitoes per group, according to the method described below. These samples were not dissected.

ZIKV and *Wolbachia* RT-qPCR analysis

Whole mosquito samples were cut into two parts: head/thorax, and abdomen, and these were homogenized as previously described, and processed independently (Moreira et al., 2009). RNA was extracted from mosquito tissues using the High Pure Viral Nucleic Acid Kit (Roche) following manufacturer's instructions. RNA was extracted directly from individual saliva samples using the same protocol, but half the volume of each reagent. RNA samples were diluted to 50 ng/µL in nuclease-free water, and stored at -

80ºC. ZIKV levels in these samples were then quantified by RT-qPCR using a LightCycler® 96 instrument (Roche) and previously described primers and probe (ZIKV 835; ZIKV911c – ZIKV 860-FAM) (Lanciotti et al., 2008). *Wolbachia* levels were quantified for all *w*Mel_Br samples using the *Wolbachia WD0513* gene, a constitutively expressed transposable element (Ferguson et al., 2015). Thermocycling conditions were as follows: an initial reverse transcription step at 50ºC for 5 min; RT inactivation/initial denaturation at 95ºC for 20 sec, and 40 cycles of 95ºC for 3 sec and 60ºC for 30 sec. The total reaction volume was 10 µL (4x TaqMan Fast Virus 1-Step Master Mix (ThermoFisher), 1 µM primers and probe, and 125ng of RNA template).
Each sample was run in duplicate for ZIKV or *WD0513*, and *Ae. aegypti* Ribosomal S17 (*rps17*), which served as a reaction control (Moreira et al., 2009). Samples were analyzed using absolute quantification, by comparison to serial dilutions of either gene product, cloned and amplified in the pGEMT-Easy plasmid (Promega), according to manufacturer's instructions. Negative control samples were normalized between plates, and were used as reference to determine a minimum threshold for positive samples. ZIKV or *Wolbachia* load data were calculated as the total number of copies per tissue or saliva sample.

Statistical Analysis

ZIKV prevalence in mosquito tissues and saliva samples were compared using Fisher's exact test, and infection intensity data were compared using Mann Whitney U test, both using Prism V6 (Graphpad) (Tables S1-S3). *Wolbachia* density data were compared across ZIKV-infected/uninfected *w*Mel_Br mosquitoes for both ZIKV isolates through permutational multivariate analysis of variance (PERMANOVA) (Table S4), via the *adonis()* function in R, through the GUSTA ME interface (mb3is.megx.net/gustame) (Buttigieg and Ramette, 2014). Spearman correlation was used to determine if there was a relationship between ZIKV and *WD0513* levels in ZIKV-infected *w*Mel_Br mosquitoes (Prism V6).

Supplemental References:

ANDERSON, S. L., RICHARDS, S. L. & SMARTT, C. T. 2010. A simple method for determining arbovirus transmission in mosquitoes. *J Am Mosq Control Assoc,* 26**,** 108-11.

BUTTIGIEG, P. L. & RAMETTE, A. 2014. A guide to statistical analysis in microbial ecology: a community-focused, living review of multivariate data analyses. *FEMS Microbiol Ecol,* 90**,** 543-50.

DUTRA, H. L., DOS SANTOS, L. M., CARAGATA, E. P., SILVA, J. B., VILLELA, D. A., MACIEL-DE-FREITAS, R. & MOREIRA, L. A. 2015. From lab to field: the influence of urban landscapes on the invasive potential of *Wolbachia* in Brazilian *Aedes aegypti* mosquitoes. *PLoS Negl Trop Dis,* 9**,** e0003689.

HAMEL, R., DEJARNAC, O., WICHIT, S., EKCHARIYAWAT, P., NEYRET, A., LUPLERTLOP, N., PERERA-LECOIN, M., SURASOMBATPATTANA, P., TALIGNANI, L., THOMAS, F., et al. 2015. Biology of Zika virus infection in Human skin cells. *J Virol,* 89**,** 8880-96.

LANCIOTTI, R. S., KOSOY, O. L., LAVEN, J. J., VELEZ, J. O., LAMBERT, A. J., JOHNSON, A. J., STANFIELD, S. M. & DUFFY, M. R. 2008. Genetic and serologic properties of Zika virus associated with an epidemic, Yap State, Micronesia, 2007. *Emerg Infect Dis,* 14**,** 1232-9.
