# Supplementary material for: Wolbachia Blocks Currently Circulating Zika Virus Isolates in Brazilian Aedes aegypti Mosquitoes
Source: Cell Host Microbe. 2016 Jun 8;19(6):771–4. doi: 10.1016/j.chom.2016.04.021 (PMC4906366; doi:10.1016/j.chom.2016.04.021)
Supplement: Document S2. Article plus Supplemental Information [file mmc2.pdf]

Brief Report

Cell Host & Microbe

Wolbachia Blocks Currently Circulating Zika Virus Isolates in Brazilian *Aedes aegypti* Mosquitoes

Graphical Abstract

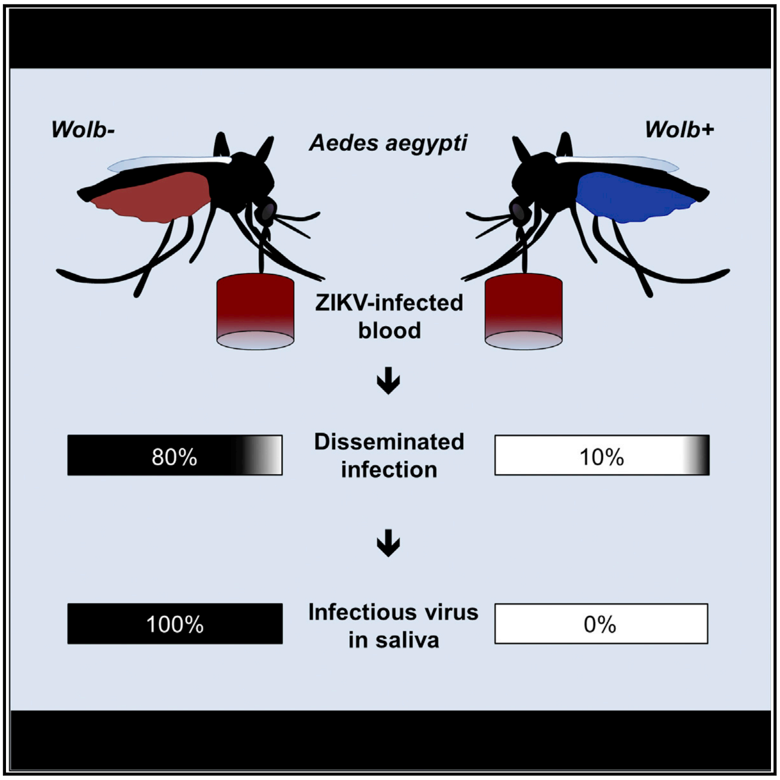

Highlights

- Mosquitoes harboring *Wolbachia* were resistant to current circulating Zika virus isolates
- Zika virus prevalence, intensity, and disseminated infection were reduced
- Saliva from *Wolbachia*-harboring mosquitoes did not contain infectious Zika virus

Authors

Heverton Leandro Carneiro Dutra, Marcele Neves Rocha, Fernando Braga Stehling Dias, Simone Brutman Mansur, Eric Pearce Caragata, Luciano Andrade Moreira

Correspondence

luciano@cpqrr.fiocruz.br

In Brief

Strategies to combat Zika virus (ZIKV) and its mosquito vector are urgently needed. Dutra et al. report that *Wolbachia*-carrying mosquitoes are highly resistant to ZIKV and display reduced virus prevalence and intensity. Saliva from *Wolbachia*-carrying mosquitoes did not contain infectious virus, suggesting the possibility to block ZIKV transmission.

Dutra et al., 2016, Cell Host & Microbe *19*, 771–774
June 8, 2016 © 2016 The Author(s). Published by Elsevier Inc.
http://dx.doi.org/10.1016/j.chom.2016.04.021

CellPress

Wolbachia Blocks Currently Circulating Zika Virus Isolates in Brazilian *Aedes aegypti* Mosquitoes

Heverton Leandro Carneiro Dutra,[1] Marcele Neves Rocha,[1] Fernando Braga Stehling Dias,[1,2] Simone Brutman Mansur,[1] Eric Pearce Caragata,[1] and Luciano Andrade Moreira[1,*]
[1]Mosquitos Vetores: Endossimbiontes e Interação Patógeno-Vetor, Centro de Pesquisas René Rachou—Fiocruz, Belo Horizonte, MG, 30190-002, Brazil
[2]Plataforma de Vetores de Doenças—Fiocruz, CE, 60190-800, Brazil
*Correspondence: luciano@cpqrr.fiocruz.br
http://dx.doi.org/10.1016/j.chom.2016.04.021

SUMMARY

The recent association of Zika virus with cases of microcephaly has sparked a global health crisis and highlighted the need for mechanisms to combat the Zika vector, *Aedes aegypti* mosquitoes. *Wolbachia pipientis*, a bacterial endosymbiont of insect, has recently garnered attention as a mechanism for arbovirus control. Here we report that *Aedes aegypti* harboring *Wolbachia* are highly resistant to infection with two currently circulating Zika virus isolates from the recent Brazilian epidemic. *Wolbachia*-harboring mosquitoes displayed lower viral prevalence and intensity and decreased disseminated infection and, critically, did not carry infectious virus in the saliva, suggesting that viral transmission was blocked. Our data indicate that the use of *Wolbachia*-harboring mosquitoes could represent an effective mechanism to reduce Zika virus transmission and should be included as part of Zika control strategies.

The mosquito *Aedes aegypti*, typically linked with dengue (*Flaviviridae*) (Kyle and Harris, 2008) and chikungunya (*Togaviridae*) (Morrison, 2014) transmission, is also associated with the alarming spread of Zika virus (ZIKV) (*Flaviviridae*), a previously obscure arbovirus that has recently gone global (Enserink, 2015). Since 2007, ZIKV infection has been reported in 39 countries worldwide (Martínez de Salazar et al., 2016), including Brazil, where infection was first linked to cases of microcephaly during a large outbreak in 2015 (Mlakar et al., 2016; Oliveira Melo et al., 2016). Combined with the implication of the virus in cases of the autoimmune disorder Guillain-Barré syndrome (Araujo et al., 2016), ZIKV has ballooned into a public health crisis.

In the absence of a vaccine, current effective control options are limited to reducing the abundance of mosquito vector populations (Heintze et al., 2007). However, there is a clear need for novel efficacious approaches, given that existing strategies such as insecticides (Maciel-de-Freitas et al., 2014) and larval biological control (Vu et al., 2005) have proven unsustainable and ineffective at halting disease spread (Kyle and Harris, 2008).

After decades of being proposed as a potential means of vector control, the endosymbiotic bacterium *Wolbachia*, present in an estimated 40% of all known terrestrial insect species (Zug and Hammerstein, 2012), is currently being utilized around the world as part of an innovative approach to control the transmission of dengue (http://www.eliminatedengue.com) and other pathogens (Bourtzis et al., 2014). This is possible because the reproductive parasitism associated with *Wolbachia* infection, typified by cytoplasmic incompatibility (Werren et al., 2008), gives the bacterium the ability to quickly and stably invade host populations (Hoffmann et al., 2011). Critically, the bacterium also blocks the transmission of many important human pathogens in mosquitoes, including *Plasmodium* and chikungunya (Bian et al., 2013; Caragata et al., 2016; Moreira et al., 2009), giving it great utility as a control agent.

As many different strains of the bacterium cause this inhibition, we hypothesized that the *w*Mel *Wolbachia* strain (*w*Mel_Br), currently being utilized as part of dengue control efforts in Brazil, might be able to restrict ZIKV infection and transmission in *Ae. aegypti*. To that end, we performed experimental infections with two currently circulating ZIKV isolates and used a qRT-PCR-based assay to a quantify ZIKV levels in mosquito tissues and saliva, in order to assess whether *Wolbachia* could potentially be used to combat the emerging Zika pandemic.

Through experimental infection and transmission assays using two currently circulating Brazilian ZIKV isolates (BRPE243/2015 [BRPE] and SPH/2015 [SPH]) (Faria et al., 2016), we compared ZIKV infection in *w*Mel-infected mosquitoes (*w*Mel_Br) with *Wolbachia*-uninfected mosquitoes collected in Urca, Rio de Janeiro, Brazil in early 2016 (Br). Due to the regular introduction of F_1 Br males (the eggs of field-collected Br mosquitoes) in *w*Mel_Br colony cages over 2 years, both lines had a similar genetic background (see Supplemental Experimental Procedures).

The ZIKVs were isolated in the field in late 2015 and maintained in cell culture, and viral titers were quantified via plaque-forming assay prior to experimental infection (Table 1). In two separate experiments, fresh ZIKV-infected supernatant was harvested from culture, mixed with human blood, and used to orally infect *w*Mel_Br and Br mosquitoes. ZIKV levels were quantified in mosquito heads/thoraces and in abdomens at 7 and 14 days post-infection (dpi) using a TaqMan-based qRT-PCR assay (Figure 1).

The prevalence of ZIKV infection was significantly reduced among *Wolbachia*-infected mosquitoes (Table 1, analysis via Fisher's exact test, $p < 0.0001$ unless stated). For the BRPE isolate (Figure 1A), *Wolbachia* decreased ZIKV prevalence by 35% in abdomens, although there was no significant difference

 © 2016 The Author(s). Published by Elsevier Inc.
This is an open access article under the CC BY license (http://creativecommons.org/licenses/by/4.0/).

Table 1. Effects of *Wolbachia* on ZIKV Prevalence

Isolate	ZIKV Titer (PFU/mL)	Days Post-infection	Head/Thorax Infection Rate *w*Mel_Br	Head/Thorax Infection Rate Br	Abdomen Infection Rate *w*Mel_Br	Abdomen Infection Rate Br	Saliva Infection Rate *w*Mel_Br	Saliva Infection Rate Br
BRPE	5.0×10^6	7	0	65	55	85	–	–
		14	10	100	35	100	45	100
SPH	8.7×10^3	7	5	95	30	90	–	–
		14	25	95	30	95	–	–

Ae. aegypti were orally infected with fresh, low-passage ZIKV. Initial viral titer was determined by plaque-forming assay. Saliva infection was only examined for mosquitoes at 14 days post-infection with the BRPE isolate. Infection rates are given as percentages. n = 20 per group unless specified. ZIKV, Zika virus; PFU, plaque-forming units; BRPE, ZIKV/*H. sapiens*/Brazil/BRPE243/2015; SPH, ZIKV/*H. sapiens*/Brazil/SPH/2015; *w*Mel_Br, *Wolbachia*-infected; Br, *Wolbachia*-uninfected.

for this tissue ($p > 0.05$), by 100% in head/thoraces at 7 dpi, and by 65% and 90% at 14 dpi, respectively. For the SPH isolate (Figure 1B), *Wolbachia* reduced prevalence by 95% and 67% in head/thoraces and abdomens ($p = 0.0002$), respectively, at 7 dpi, and by 74% and 68% in head/thoraces and abdomens, respectively, at 14 dpi.

Likewise, the intensity of ZIKV infection was greatly reduced in *w*Mel_Br mosquitoes for both tissues and time points (Mann-Whitney U tests, $p < 0.0001$). Additionally, we observed that median ZIKV titers in the head/thoraces of Br mosquitoes increased over time for both isolates (Mann-Whitney U test; BRPE, $p < 0.0001$; SPH, $p = 0.0094$), while there was no such effect in *w*Mel_Br mosquitoes.

Saliva was collected from Br and *w*Mel_Br mosquitoes at 14 dpi, after the 5- to 10-day ZIKV extrinsic incubation period was likely completed (Li et al., 2012), in order to determine if *Wolbachia* infection also inhibited ZIKV transmission (Figure 1C). We used mosquitoes infected with the BRPE isolate as it had a higher titer in culture (Table 1). ZIKV levels were quantified directly for individual saliva samples using the same qRT-PCR assay. We observed that *Wolbachia* infection reduced ZIKV prevalence in individual saliva samples by 55% (Fisher's exact test, $p < 0.0001$) and median ZIKV copies by approximately 5 logs (Mann-Whitney U test, $p < 0.0001$).

To determine if the virus in these samples was infectious, a further ten *w*Mel_Br and ten Br saliva samples, from the samples described above, were intrathoracically injected into 8–14 naive Br mosquitoes each (Figure 1D), using a previously described method (Ferguson et al., 2015). The overall mortality rate among injected mosquitoes was 11.93%. The presence or absence of ZIKV infection was determined at 5 dpi in eight mosquitoes injected with each saliva, amounting to a mean proportion sampled of 0.68. Of the 80 mosquitoes injected with Br saliva, 68 (85%) became infected with ZIKV, with all Br saliva samples producing at least one infected mosquito. In contrast, none of the 80 mosquitoes injected with *w*Mel_Br saliva became infected (Fisher's exact test, $p < 0.0001$; odds ratio 882.3, 95% CI, 51.3–15187), indicating that while some of the *w*Mel_Br saliva samples did contain detectable ZIKV, we saw no evidence that the saliva contained infectious virus.

There is a clear correlation between the inhibition of pathogens by *Wolbachia* and bacterial density in insect tissues (Joubert et al., 2016; Martinez et al., 2014). In order to determine if there was a link between *Wolbachia* density and ZIKV prevalence and intensity, we measured total *Wolbachia* RNA levels in the *w*Mel_Br mosquitoes used in the ZIKV infection assays, using qRT-PCR as described above. We saw that ZIKV infection explained less than 5% of the variance in *Wolbachia* density that was observed between ZIKV-infected and -uninfected *w*Mel_Br mosquitoes at either 7 dpi or 14 dpi and was not a significant predictor (PERMANOVA; $p > 0.05$). Furthermore, we observed no relationship between *Wolbachia* density and ZIKV load among *w*Mel_Br mosquitoes that became infected with the virus (Spearman correlation; heads/thoraces, $r = 0.5952$, $p = 0.1323$; abdomens, $r = -0.01891$, $p = 0.9210$). This suggests that there may not be a direct link between *Wolbachia* density in individual mosquitoes and ZIKV infection, indicating that the inhibition of ZIKV may arise through other means, indirectly due to the presence of the bacterium (Caragata et al., 2013; Moreira et al., 2009; Pan et al., 2012; Rancès et al., 2012).

Our results indicate that the ability of *Wolbachia* infection to greatly reduce the capacity of mosquitoes to harbor and transmit a range of medically important pathogens, including the dengue and chikungunya viruses (Caragata et al., 2016; Moreira et al., 2009; Walker et al., 2011) also extends to ZIKV. While *w*Mel did not completely inhibit ZIKV infection, we observed a similar decrease in prevalence and intensity of infection to that of *w*Mel-infected *Ae. aegypti* challenged with viremic blood from dengue patients, which was considered sufficient to drastically decrease viral transmission (Ferguson et al., 2015). Additionally, the fact that we did not observe an increase in disseminated ZIKV infection over time, and that ZIKV prevalence and infectivity in *w*Mel_Br mosquito saliva was significantly decreased, may indicate that, as for dengue, *w*Mel extends the ZIKV extrinsic incubation period (Ye et al., 2015). This in turn would likely further decrease overall ZIKV transmission rates, given the small decrease in lifespan associated with *w*Mel infection (Walker et al., 2011).

We observed that the *w*Mel *Wolbachia* infection in *Ae. aegypti* greatly inhibited ZIKV infection in mosquito abdomens, and it reduced disseminated infection in heads and thoraces and ZIKV prevalence in mosquito saliva. Most critically, our results suggest that saliva from *w*Mel-infected mosquitoes did not contain infectious virus. That this inhibition occurred for two ZIKV isolates that circulated in Brazil during the 2015 epidemic, and for mosquitoes with a wild-type genetic background, suggests that *w*Mel could greatly reduce ZIKV transmission in field populations of *Ae. aegypti*, which in turn would likely reduce the frequency of Zika-associated pathology in humans.

Wolbachia can invade and persist in wild mosquito populations (Hoffmann et al., 2014) and represents a relatively

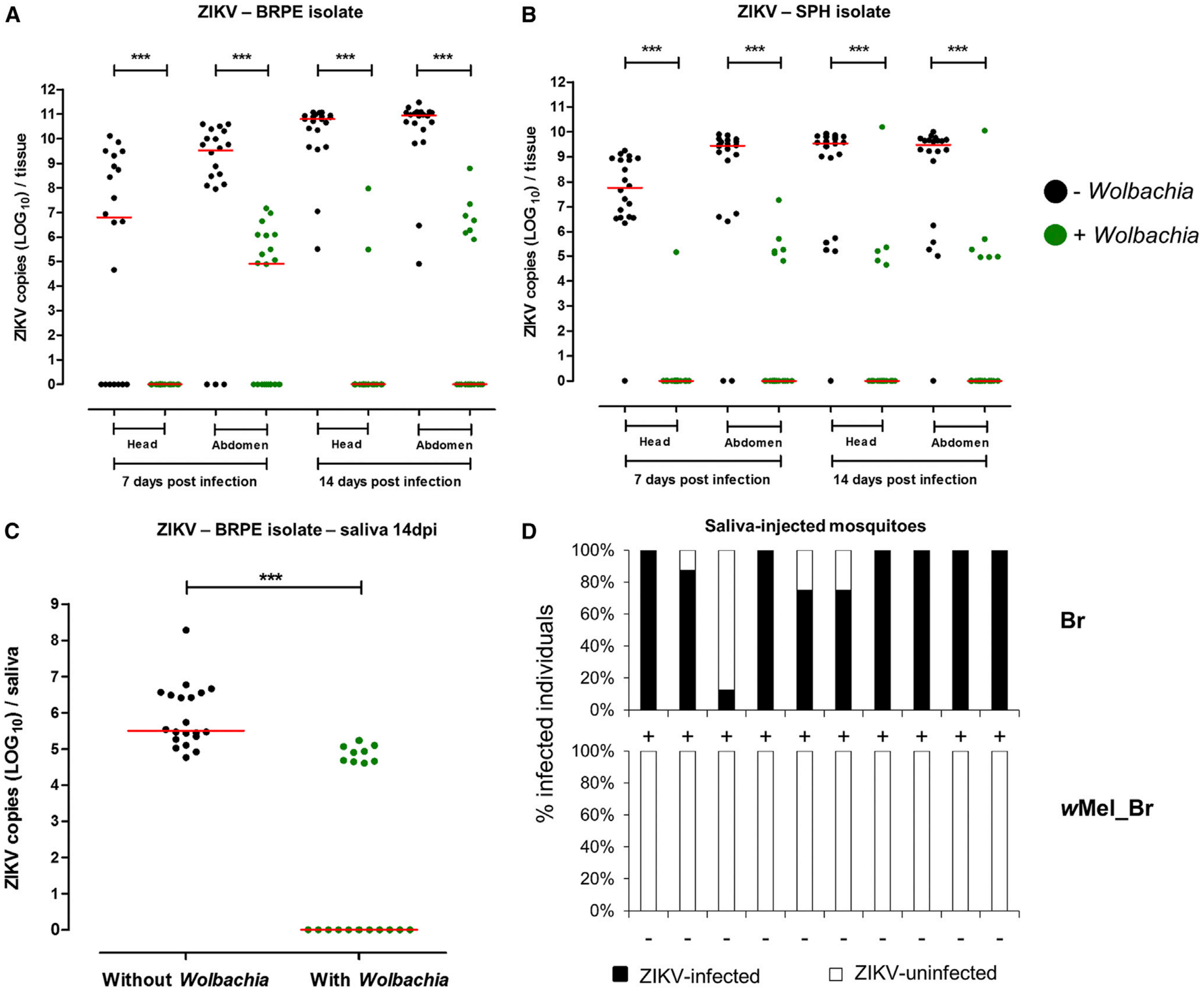

Figure 1. *Wolbachia* Infection Restricts ZIKV Infection in *Ae. aegypti* Mosquitoes

(A–C) *Wolbachia*-infected (green circles) and -uninfected (black circles) mosquitoes were orally challenged with either (A) the BRPE or (B) the SPH ZIKV isolates. *Wolbachia* infection reduced both prevalence and intensity of ZIKV infection in mosquito heads/thoraces and abdomens at 7 and 14 dpi. Saliva was then collected for mosquitoes infected with the BRPE ZIKV isolate at 14 dpi infection (C), and we observed that saliva from *Wolbachia*-infected mosquitoes had a significantly lower rate of saliva infection and median viral load.

(D) When these saliva samples were injected into ZIKV-uninfected Br mosquitoes, all of the Br saliva samples contained infectious virus, while no *w*Mel_Br saliva produced a subsequent infection (columns: black, percentage infected; white, percentage uninfected; +, saliva contained infectious virus, –, saliva did not contain infectious virus). Absolute ZIKV copy numbers were quantified via qRT-PCR.

In (A)–(C), each circle represents tissue or saliva from a single adult female (n = 20 per group). Red lines indicate the median ZIKV copies. ***, $p < 0.0001$; analysis by Mann-Whitney U test. In (D), each column represents mosquitoes injected with a single saliva sample.

low-cost, self-sustaining form of mosquito control that is already being trialed in countries where ZIKV outbreaks have been reported and has recently been recommended by the World Health Organization as a suitable tool to control ZIKV transmission (http://migre.me/tDWVe). It is important to point out that extensive public engagement will be required before releases of *Wolbachia*-infected mosquitoes can be scaled up for use in other areas. However, the results presented here indicate that *w*Mel-infected *Ae. aegypti* represent a realistic and effective option to combat the ZIKV burden in Brazil and potentially in other countries and should be considered as an integral part of future control efforts.

The work reported in this paper was performed under the oversight of the Committee for Ethics in Research (CEP)/FIOCRUZ (License CEP 732.621).

SUPPLEMENTAL INFORMATION

Supplemental Information includes Supplemental Experimental Procedures and four tables and can be found with this article online at http://dx.doi.org/10.1016/j.chom.2016.04.021.

AUTHOR CONTRIBUTIONS

Conceptualization, H.L.C.D., M.N.R., and L.A.M.; Methodology, H.L.C.D. F.B.S.D., E.P.C., and L.A.M.; Formal analysis, H.L.C.D. and E.P.C.; Investigation, H.L.C.D.; M.N.R., F.B.S.D., S.B.M., and E.P.C.; Writing—Original Draft, H.L.C.D.; Writing—Review & Editing, H.L.C.D., E.P.C., and L.A.M.; Funding Acquisition, L.A.M; Resources, L.A.M.; Supervision, L.A.M.

ACKNOWLEDGMENTS

We are grateful to all members of the Mosquitos Vetores Group (MV—CPqRR/FIOCRUZ), particularly Jéssica Silva, who helped to develop the salivation assay. We thank Dr. Luis Villegas for helpful discussion on statistics and Dr. Alexandre Machado for assistance with viral cultures. The Zika virus isolates were kindly provided by the Department of Virology and Experimental Therapy (Aggeu Magalhães Research Center/FIOCRUZ) and by the Laboratory of Viral Isolation (Evandro Chagas Institute). We thank INCT-EM for the Real-Time PCR machine, and the Brazilian and Australian teams of the Eliminate Dengue program, particularly Prof. Scott L. O'Neill for donating the original *w*Mel line and the Entomology team for providing field mosquito eggs. This work was supported by FAPEMIG, CNPq, CAPES, the Brazilian Ministry of Health (DECIT/SVS), and a grant to Monash University from the Foundation for the National Institutes of Health through the Vector-Based Transmission of Control: Discovery Research (VCTR) program of the Grand Challenges in Global Health Initiatives of the Bill and Melinda Gates Foundation.

Received: April 15, 2016
Revised: April 26, 2016
Accepted: April 26, 2016
Published: May 4, 2016

REFERENCES

Araujo, L.M., Ferreira, M.L., and Nascimento, O.J. (2016). Guillain-Barré syndrome associated with the Zika virus outbreak in Brazil. Arq. Neuropsiquiatr. *74*, 253–255.

Bian, G., Joshi, D., Dong, Y., Lu, P., Zhou, G., Pan, X., Xu, Y., Dimopoulos, G., and Xi, Z. (2013). *Wolbachia* invades *Anopheles stephensi* populations and induces refractoriness to *Plasmodium* infection. Science *340*, 748–751.

Bourtzis, K., Dobson, S.L., Xi, Z., Rasgon, J.L., Calvitti, M., Moreira, L.A., Bossin, H.C., Moretti, R., Baton, L.A., Hughes, G.L., et al. (2014). Harnessing mosquito-*Wolbachia* symbiosis for vector and disease control. Acta Trop. *132* (*Suppl*), S150–S163.

Caragata, E.P., Rancès, E., Hedges, L.M., Gofton, A.W., Johnson, K.N., O'Neill, S.L., and McGraw, E.A. (2013). Dietary cholesterol modulates pathogen blocking by *Wolbachia*. PLoS Pathog. *9*, e1003459.

Caragata, E.P., Dutra, H.L., and Moreira, L.A. (2016). Exploiting intimate relationships: Controlling mosquito-transmitted disease with *Wolbachia*. Trends Parasitol. *32*, 207–218.

Enserink, M. (2015). INFECTIOUS DISEASES. An obscure mosquito-borne disease goes global. Science *350*, 1012–1013.

Faria, N.R., Azevedo, Rdo.S., Kraemer, M.U., Souza, R., Cunha, M.S., Hill, S.C., Thézé, J., Bonsall, M.B., Bowden, T.A., Rissanen, I., et al. (2016). Zika virus in the Americas: Early epidemiological and genetic findings. Science *352*, 345–349.

Ferguson, N.M., Kien, D.T., Clapham, H., Aguas, R., Trung, V.T., Chau, T.N., Popovici, J., Ryan, P.A., O'Neill, S.L., McGraw, E.A., et al. (2015). Modeling the impact on virus transmission of *Wolbachia*-mediated blocking of dengue virus infection of *Aedes aegypti*. Sci. Transl. Med. *7*, 279ra37.

Heintze, C., Velasco Garrido, M., and Kroeger, A. (2007). What do community-based dengue control programmes achieve? A systematic review of published evaluations. Trans. R. Soc. Trop. Med. Hyg. *101*, 317–325.

Hoffmann, A.A., Montgomery, B.L., Popovici, J., Iturbe-Ormaetxe, I., Johnson, P.H., Muzzi, F., Greenfield, M., Durkan, M., Leong, Y.S., Dong, Y., et al. (2011). Successful establishment of *Wolbachia* in *Aedes* populations to suppress dengue transmission. Nature *476*, 454–457.

Hoffmann, A.A., Iturbe-Ormaetxe, I., Callahan, A.G., Phillips, B.L., Billington, K., Axford, J.K., Montgomery, B., Turley, A.P., and O'Neill, S.L. (2014). Stability of the *w*Mel *Wolbachia* Infection following invasion into *Aedes aegypti* populations. PLoS Negl. Trop. Dis. *8*, e3115.

Joubert, D.A., Walker, T., Carrington, L.B., De Bruyne, J.T., Kien, D.H., Hoang, Nle.T., Chau, N.V., Iturbe-Ormaetxe, I., Simmons, C.P., and O'Neill, S.L. (2016). Establishment of a *Wolbachia* Superinfection in *Aedes aegypti* Mosquitoes as a Potential Approach for Future Resistance Management. PLoS Pathog. *12*, e1005434.

Kyle, J.L., and Harris, E. (2008). Global spread and persistence of dengue. Annu. Rev. Microbiol. *62*, 71–92.

Li, M.I., Wong, P.S., Ng, L.C., and Tan, C.H. (2012). Oral susceptibility of Singapore Aedes (Stegomyia) aegypti (Linnaeus) to Zika virus. PLoS Negl. Trop. Dis. *6*, e1792.

Maciel-de-Freitas, R., Avendanho, F.C., Santos, R., Sylvestre, G., Araújo, S.C., Lima, J.B., Martins, A.J., Coelho, G.E., and Valle, D. (2014). Undesirable consequences of insecticide resistance following *Aedes aegypti* control activities due to a dengue outbreak. PLoS ONE *9*, e92424.

Martinez, J., Longdon, B., Bauer, S., Chan, Y.S., Miller, W.J., Bourtzis, K., Teixeira, L., and Jiggins, F.M. (2014). Symbionts commonly provide broad spectrum resistance to viruses in insects: a comparative analysis of *Wolbachia* strains. PLoS Pathog. *10*, e1004369.

Martínez de Salazar, P., Suy, A., Sánchez-Montalvá, A., Rodó, C., Salvador, F., and Molina, I. (2016). Zika fever. Enferm. Infecc. Microbiol. Clin. *34*, 247–252.

Mlakar, J., Korva, M., Tul, N., Popović, M., Poljšak-Prijatelj, M., Mraz, J., Kolenc, M., Resman Rus, K., Vesnaver Vipotnik, T., Fabjan Vodušek, V., et al. (2016). Zika Virus Associated with Microcephaly. N. Engl. J. Med. *374*, 951–958.

Moreira, L.A., Iturbe-Ormaetxe, I., Jeffery, J.A., Lu, G., Pyke, A.T., Hedges, L.M., Rocha, B.C., Hall-Mendelin, S., Day, A., Riegler, M., et al. (2009). A *Wolbachia* symbiont in *Aedes aegypti* limits infection with dengue, Chikungunya, and *Plasmodium*. Cell *139*, 1268–1278.

Morrison, T.E. (2014). Reemergence of chikungunya virus. J. Virol. *88*, 11644–11647.

Oliveira Melo, A.S., Malinger, G., Ximenes, R., Szejnfeld, P.O., Alves Sampaio, S., and Bispo de Filippis, A.M. (2016). Zika virus intrauterine infection causes fetal brain abnormality and microcephaly: tip of the iceberg? Ultrasound Obstet. Gynecol. *47*, 6–7.

Pan, X., Zhou, G., Wu, J., Bian, G., Lu, P., Raikhel, A.S., and Xi, Z. (2012). *Wolbachia* induces reactive oxygen species (ROS)-dependent activation of the Toll pathway to control dengue virus in the mosquito *Aedes aegypti*. Proc. Natl. Acad. Sci. USA *109*, E23–E31.

Rancès, E., Ye, Y.H., Woolfit, M., McGraw, E.A., and O'Neill, S.L. (2012). The relative importance of innate immune priming in *Wolbachia*-mediated dengue interference. PLoS Pathog. *8*, e1002548.

Vu, S.N., Nguyen, T.Y., Tran, V.P., Truong, U.N., Le, Q.M., Le, V.L., Le, T.N., Bektas, A., Briscombe, A., Aaskov, J.G., et al. (2005). Elimination of dengue by community programs using Mesocyclops(Copepoda) against *Aedes aegypti* in central Vietnam. Am. J. Trop. Med. Hyg. *72*, 67–73.

Walker, T., Johnson, P.H., Moreira, L.A., Iturbe-Ormaetxe, I., Frentiu, F.D., Mcmeniman, C.J., Leong, Y.S., Dong, Y., Axford, J., Kriesner, P., et al. (2011). The wMel Wolbachia strain blocks dengue and invades caged Aedes aegypti populations. Nature *476*, 450–455.

Werren, J.H., Baldo, L., and Clark, M.E. (2008). *Wolbachia*: master manipulators of invertebrate biology. Nat. Rev. Microbiol. *6*, 741–751.

Ye, Y.H., Carrasco, A.M., Frentiu, F.D., Chenoweth, S.F., Beebe, N.W., van den Hurk, A.F., Simmons, C.P., O'Neill, S.L., and McGraw, E.A. (2015). *Wolbachia* reduces the transmission potential of dengue-infected *Aedes aegypti*. PLoS Negl. Trop. Dis. *9*, e0003894.

Zug, R., and Hammerstein, P. (2012). Still a host of hosts for *Wolbachia*: analysis of recent data suggests that 40% of terrestrial arthropod species are infected. PLoS ONE *7*, e38544.

Cell Host & Microbe, Volume *19*

Supplemental Information

Wolbachia Blocks Currently Circulating Zika Virus Isolates in Brazilian *Aedes aegypti* Mosquitoes

Heverton Leandro Carneiro Dutra, Marcele Neves Rocha, Fernando Braga Stehling Dias, Simone Brutman Mansur, Eric Pearce Caragata, and Luciano Andrade Moreira

Table S1, related to Figure 1. Statistical output for comparison of ZIKV levels in *Wolbachia*-infected and uninfected mosquito tissues

***w*Mel_Br vs Br**	**Infection prevalence: Fisher's exact test**	**Infection intensity: Mann Whitney U test**
**BRPE**		
7dpi heads/thoraces	$P < 0.0001$	$U = 70.00$, $P < 0.0001$
7dpi abdomens	$P = 0.0824$	$U = 46.50$, $P < 0.0001$
14dpi heads/thoraces	$P < 0.0001$	$U = 2.00$, $P < 0.0001$
14dpi abdomens	$P < 0.0001$	$U = 11.00$, $P < 0.0001$
**SPH**		
7dpi heads/thoraces	$P < 0.0001$	$U = 10.50$, $P < 0.0001$
7dpi abdomens	$P = 0.0002$	$U = 29.00$, $P < 0.0001$
14dpi heads/thoraces	$P < 0.0001$	$U = 34.50$, $P < 0.0001$
14dpi abdomens	$P < 0.0001$	$U = 37.00$, $P < 0.0001$

Abbreviations: 7/14dpi - 7/14 days post infection. BRPE - ZIKV/*H. sapiens*/Brazil/BRPE243/2015, SPH - ZIKV/*H. sapiens*/Brazil/SPH/2015, *w*Mel_Br - *Wolbachia*-infected *Ae. aegypti*, Br - *Wolbachia*-uninfected *Ae. aegypti*.

Table S2, related to Figure 1. Statistical output for comparison of ZIKV levels in mosquito tissues over time.

**7dpi vs 14dpi**	**Mann Whitney U test**
**BRPE**	
*w*Mel_Br heads/thoraces	$U = 180.0$, $P = 0.1626$
wMel_Br abdomens	$U = 182.5$, $P = 0.6146$
Br heads/thoraces	$U = 27.00$, $P < 0.0001$
Br abdomens	$U = 52.00$, $P < 0.0001$
**SPH**	
*w*Mel_Br heads/thoraces	$U = 159.5$, $P = 0.0816$
wMel_Br abdomens	$U = 199.0$, $P = 0.9867$
Br heads/thoraces	$U = 103.5$, $P = 0.0094$
Br abdomens	$U = 189.0$, $P = 0.7764$

Abbreviations: 7/14dpi - 7/14 days post infection. BRPE - ZIKV/*H. sapiens*/Brazil/BRPE243/2015, SPH - ZIKV/*H. sapiens*/Brazil/SPH/2015, *w*Mel_Br - *Wolbachia*-infected *Ae. aegypti*, Br - *Wolbachia*-uninfected *Ae. aegypti*.

Table S3, related to Figure 1. Statistical output for comparison of ZIKV levels in the saliva of *Wolbachia*-infected and -uninfected mosquitoes

***w*Mel_Br vs Br saliva**	**Infection prevalence: Fisher's exact test**	**Infection intensity: Mann Whitney U test**
Individual saliva samples	$P = 0.0001$	$U = 13.00$, $P < 0.0001$
Saliva-injected mosquitoes	$P < 0.0001$	NA

Abbreviations: *w*Mel_Br - *Wolbachia*-infected *Ae. aegypti*, Br - *Wolbachia*-uninfected *Ae. aegypti*.

Table S4, related to Figure 1. Statistical output for comparison of *Wolbachia* density amongst ZIKV-infected and -uninfected *w*Mel_Br mosquitoes

PREMANOVA

**7dpi**	**df**	**SS**	**MS**	***F***	R^2	**Pr(>F)**
ZIKV infection	1	2.61E+14	2.61E+14	1.8286	0.04434	0.186
ZIKV isolate	1	6.31E+14	6.31E+14	4.4139	0.10702	0.029
Residuals	35	5.00E+15	1.43E+14		0.84864	
Total	37	5.90E+15			1.00000	

**14dpi**	**df**	**SS**	**MS**	***F***	R^2	**Pr(>F)**
ZIKV infection	1	5.63E+13	5.63E+13	0.1188	0.00289	0.813
ZIKV isolate	1	1.85E+15	1.85E+15	3.9087	0.09527	0.053
Residuals	37	1.76E+16	4.74E+14		0.90184	
Total	39	1.95E+16			1.00000	

Abbreviations: ZIKV - Zika virus, 7/14dpi - 7/14 days post infection.

Supplemental Experimental Procedures

Mosquito rearing

All experiments involved two *Ae. aegypti* lines. The first (*w*Mel_Br) was generated by introducing the *w*Mel *Wolbachia* strain into a Brazilian genetic background through backcrossing (Dutra et al., 2015). Experiments were performed 35 generations after the initial backcrossing. The second, (Br) was an F_1 wild-type line derived from material collected from ovitraps in the suburb of Urca, RJ, Brazil at the beginning of 2016. This line never had any contact with *Wolbachia*-infected mosquitoes. For 25 generations prior to experimentation, 200 F_1-F_2 Br males for every 600 *w*Mel_Br females were introduced into *w*Mel_Br colony cages each generation to prevent inbreeding effects, and maintain a similar genetic background between the two lines. All mosquitoes were maintained in a climate-controlled insectary under previously described conditions (Dutra et al., 2015).

ZIKV isolation and culture

The Zika viruses used in this work were isolated in 2015 from human serum collected from two symptomatic patients, the first one from Recife, PE, in northeastern Brazil (ZIKV/*H. sapiens*/Brazil/BRPE243/2015), and the second from Sumaré, SP, in the Southeast of the country (ZIKV/*H. sapiens*/Brazil/SPH/2015) (Faria et al., 2016). Virus stocks were passaged in *Aedes albopictus* cell line (C6/36) grown in Leibowitz L-15 medium supplemented with 10% fetal bovine serum (FBS), 1% penicillin/streptomycin (Gibco), and maintained at 28ºC, as previously described (Hamel et al., 2015). Fresh supernatant from infected C6/36 cells was harvested 7 days after infection with a corresponding viral titer of $5x10^6$ PFU/mL for the BRPE isolate, and $8.7x10^3$ PFU/mL for the SPH isolate, and used to orally infect mosquitoes without ever being frozen.

Infection of mosquitoes with ZIKV

ZIKV was collected from C6/36 cell culture supernatant and then re-suspended 2:1 in fresh whole human blood. Four days-old adult female mosquitoes were starved for 24 hrs prior to feeding, and allowed to feed on the blood-virus mixtures for 1 hr using glass feeders covered with pig intestine as a membrane, and maintained at 37°C using a water bath. After feeding, mosquitoes that were not fully engorged were removed. Mosquitoes were collected at both 7dpi and 14dpi, and stored at -80ºC until processing.

Saliva collection

Individual mosquito saliva was collected at 14 days post-infection from mosquitoes infected with the BRPE ZIKV isolate according to a previously published protocol, with some modifications (Anderson et al., 2010). Briefly, mosquitoes were starved overnight prior to harvesting. On collection day, mosquitoes were knocked down with CO_2, and kept at 4ºC while legs and wings were removed. Each mosquito's proboscis was inserted into a sterile, filtered 10μL pipette tip containing 5μL of a 1:1 solution of sterile fetal bovine serum: 30% sucrose, and allowed to salivate for 30 minutes. Mosquitoes were then visually verified to be alive by checking for movement. The contents of the tips were then collected in sterile 0.5mL tubes and stored at -80ºC prior to processing. One third of the saliva samples were used for injections while the remainder were used for direct quantification.

Confirmation of saliva ZIKV infectivity

Female Br mosquitoes were injected intrathoracically with saliva collected from ZIKV-infected *w*Mel_Br or Br females, in order to determine if the saliva contained infectious virus. Mosquitoes were injected using a Nanoject II hand held injector (Drummond), as previously described (Moreira et al., 2009). Each saliva sample was used to inoculate between 8-14 mosquitoes, with each receiving an average of 276nL. To avoid contamination, a fresh needle was used for each saliva. Mosquitoes were collected 5 dpi, and the presence or absence of virus was determined by RT-qPCR screening of 8 individual mosquitoes per group, according to the method described below. These samples were not dissected.

ZIKV and *Wolbachia* RT-qPCR analysis

Whole mosquito samples were cut into two parts: head/thorax, and abdomen, and these were homogenized as previously described, and processed independently (Moreira et al., 2009). RNA was extracted from mosquito tissues using the High Pure Viral Nucleic Acid Kit (Roche) following manufacturer's instructions. RNA was extracted directly from individual saliva samples using the same protocol, but half the volume of each reagent. RNA samples were diluted to 50 ng/μL in nuclease-free water, and stored at -

80ºC. ZIKV levels in these samples were then quantified by RT-qPCR using a LightCycler® 96 instrument (Roche) and previously described primers and probe (ZIKV 835; ZIKV911c – ZIKV 860-FAM) (Lanciotti et al., 2008). *Wolbachia* levels were quantified for all *w*Mel_Br samples using the *Wolbachia WD0513* gene, a constitutively expressed transposable element (Ferguson et al., 2015). Thermocycling conditions were as follows: an initial reverse transcription step at 50ºC for 5 min; RT inactivation/initial denaturation at 95ºC for 20 sec, and 40 cycles of 95ºC for 3 sec and 60ºC for 30 sec. The total reaction volume was 10 µL (4x TaqMan Fast Virus 1-Step Master Mix (ThermoFisher), 1 µM primers and probe, and 125ng of RNA template).
Each sample was run in duplicate for ZIKV or *WD0513*, and *Ae. aegypti* Ribosomal S17 (*rps17*), which served as a reaction control (Moreira et al., 2009). Samples were analyzed using absolute quantification, by comparison to serial dilutions of either gene product, cloned and amplified in the pGEMT-Easy plasmid (Promega), according to manufacturer's instructions. Negative control samples were normalized between plates, and were used as reference to determine a minimum threshold for positive samples. ZIKV or *Wolbachia* load data were calculated as the total number of copies per tissue or saliva sample.

Statistical Analysis
ZIKV prevalence in mosquito tissues and saliva samples were compared using Fisher's exact test, and infection intensity data were compared using Mann Whitney U test, both using Prism V6 (Graphpad) (Tables S1-S3). *Wolbachia* density data were compared across ZIKV-infected/uninfected *w*Mel_Br mosquitoes for both ZIKV isolates through permutational multivariate analysis of variance (PERMANOVA) (Table S4), via the *adonis()* function in R, through the GUSTA ME interface (mb3is.megx.net/gustame) (Buttigieg and Ramette, 2014). Spearman correlation was used to determine if there was a relationship between ZIKV and *WD0513* levels in ZIKV-infected *w*Mel_Br mosquitoes (Prism V6).

Supplemental References:

ANDERSON, S. L., RICHARDS, S. L. & SMARTT, C. T. 2010. A simple method for determining arbovirus transmission in mosquitoes. *J Am Mosq Control Assoc,* 26**,** 108-11.

BUTTIGIEG, P. L. & RAMETTE, A. 2014. A guide to statistical analysis in microbial ecology: a community-focused, living review of multivariate data analyses. *FEMS Microbiol Ecol,* 90**,** 543-50.

DUTRA, H. L., DOS SANTOS, L. M., CARAGATA, E. P., SILVA, J. B., VILLELA, D. A., MACIEL-DE-FREITAS, R. & MOREIRA, L. A. 2015. From lab to field: the influence of urban landscapes on the invasive potential of *Wolbachia* in Brazilian *Aedes aegypti* mosquitoes. *PLoS Negl Trop Dis,* 9**,** e0003689.

HAMEL, R., DEJARNAC, O., WICHIT, S., EKCHARIYAWAT, P., NEYRET, A., LUPLERTLOP, N., PERERA-LECOIN, M., SURASOMBATPATTANA, P., TALIGNANI, L., THOMAS, F., et al. 2015. Biology of Zika virus infection in Human skin cells. *J Virol,* 89**,** 8880-96.

LANCIOTTI, R. S., KOSOY, O. L., LAVEN, J. J., VELEZ, J. O., LAMBERT, A. J., JOHNSON, A. J., STANFIELD, S. M. & DUFFY, M. R. 2008. Genetic and serologic properties of Zika virus associated with an epidemic, Yap State, Micronesia, 2007. *Emerg Infect Dis,* 14**,** 1232-9.
